# Lift the lip: a screening guide among the dental professionals

**DOI:** 10.3389/froh.2023.1177251

**Published:** 2023-08-03

**Authors:** Sunil Babu Kotha

**Affiliations:** ^1^ Department of Preventive Dentistry, Division of Pediatric Dentistry, College of Dentistry, Riyadh Elm University (REU), Riyadh, Saudi Arabia; ^2^ Department of Pediatric and Preventive Dentistry, Sharad Pawar Dental College and Hospital, Datta Meghe Institute of Medical Sciences (Deemed to be University), Wardha, India

**Keywords:** visual tool, lip, screening guide, initial diagnosis, white spot lesions

## Introduction

Early Childhood Caries (ECC) was defined in a declaration made by the International Association for Pediatric Dentistry (IAPD) in Bangkok as the presence of one or more decayed (non-cavitated or cavitated lesions), missing or filled (due to caries) surfaces in any primary tooth of a child younger than six years of age (1). The first sign of ECC is a lesion known as a white spot, which, if left untreated, can eventually cause the enamel to crack and progress into a cavitated carious lesion. This shift is extremely rapid and takes place in a relatively short amount of time (2). Thus, early detection is essential if one has to avoid the breakdown of the teeth leading to further destruction. These carious lesions may necessitate invasive, general anesthesia-based treatments that are expensive in terms of both time and money (3).

As we all know, Prevention is better than cure. Early identification and management during the white spot condition can reverse this disease process. The first time a child goes to the dentist is a significant milestone in their life. It affords the dentist the chance to counsel parents on the prevention of oral diseases and makes it possible for the early detection of dental caries and the halting of its progression. It is suggested that the initial appointment to the dentist take place no later than 12 months of age (4). Motivating parents to participate in early intervention programs to reduce the risk of dental decay is a difficult task. However, parents take their children to pediatricians for treatment of general physical issues. Thus, it is of the utmost importance to counsel the pediatricians regarding the child's development and eruption of teeth in addition to guidance for prevention of various oral conditions at this age, in order for these children to have better oral health in the future (2, 5, 6).

Using the technique known as “Lift the lip,” the screening guide was created with the intention of assisting medical professionals in more readily recognizing the condition at a much younger age (2, 7, 8). It is a visual and nontactile method that has been created for the purpose of assessing caries and has been utilized in a number of national health and nutrition evaluation surveys (9–11). According to Begzati A et al., the presence or absence of ECC was determined via a meticulous lift-the-lip examination based on the presence of “noncavity caries/white spot lesions” or “cavity caries.” (12).

### Steps for this visual tool

Primary care practitioners are the target audience for the screening guide, which is designed to help them discover dental problems. This guide is not intended to serve as a substitute for the traditional oral examination that is carried out by an oral health expert in a dental environment (7).

**Step 1:** Raise the lip and perform a visual examination of the teeth to check for dental caries in all children younger than 5 years old: It is possible that the teeth will be in one of the following states:
  1. **Condition 1:** Enamel that is completely smooth and lustrous, free of any deposits or white spot lesions.
  2. **Condition 2:** During the examination, the early clinical sign of a white-spot lesion along the margins of the gingiva (a chalky-white appearance) can be observed. This lesion is typically coupled with sticky white deposits of food debris and plaque. If you practice good oral hygiene on a consistent basis and have a dental professional apply fluoride to your teeth, you may be able to reverse this condition and return your teeth to their healthy state.
  3. **Condition 3:** Further demineralization can range from the superficial breakdown of enamel to the irreversible formation of a brownish or black surface on the tooth, both of which are untreatable by good oral hygiene practices. Instead, it is the task of the trained professional to restore normal form and function to this condition.
**Step 2:** Informing the parent or caregiver about the status: It is necessary to provide the parent or primary caregiver with an update on the situation based on the current state of affairs.
  1. **Condition 1:** the child ought to pay a visit to the pediatric dentist once every six months.
  2. **Condition 2:** In order to prevent further damage, the parents should be instructed to make an appointment with a dentist within two to four weeks.
  3. **Condition 3:** The child must make an emergency appointment with a dentist as soon as possible in order to have the form and function of their teeth restored.
**Step 3:** Provide them with the referral form and oral health education resource.

The parent or caregiver should be given a referral form, and they should be encouraged to take action in accordance with the condition of the teeth.

## Discussion

After birth, due to the feeding patterns and lifestyle of the child, parents/caregivers should obtain adequate training to carry out secondary prevention, where the Lift-the-Lip concept is the early phase where they may spot the condition well before the destruction of the tooth (12). At this stage, the white spot, the surface is still unbroken, and the lesion beneath the surface can be reversed. In children younger than three years old, early stages of dental caries are most frequently observed on the front surface of the front teeth; hence, a routine “lift-the-lip” examination is sufficient to identify the majority of caries (13).

Pediatric primary health care physicians see a much larger number of children than their dental colleagues do, and they have the potential to play a significant part in ensuring that children who have caries receive early treatments. Every infant should have a pediatric examination that includes “lifting the lip” and inspecting the anterior maxillary incisors for signs of caries. This should be a standard part of the examination. Every normal physical examination for children should include a quick visual examination of the child's anterior teeth. This examination should take no more than a few minutes (13). Shackleton et al. employed this technique to study differences in dental caries experience in New Zealand children. The results showed that this technique was simple to carry out and only took between two and three minutes. The lift-the-lip approach offers a complete visibility of tooth surfaces, which served to influence toothbrushing as an added maneuver to remove dental plaque. This technique helped the parents and the caregivers because it enabled them to see their children's teeth more clearly (14). This “lift the lip” concept was applied by Jeniffer Curto Manrique et al. to compare two distinct techniques of tooth brushing, the modified Bass and horizontal scrub technique with and without “lift the lip,” and they came to the conclusion that lifting the lip is advantageous in not just the visual inspection, but this “lift the lip” associated with toothbrushing have the added advantage of better display of gingival one third of the teeth and the interproximal surfaces and so the cleaning (11).

It is important to point out a few of the drawbacks associated with the “lift-the-lip” assessment of younger children. The examiners could only perform a visual examination of the maxillary anterior teeth of the children who were one year old (10). In the cross-sectional study that Kaste and colleagues (10) conducted, more than 600 children (654) between the ages of 12 and 23 months were administered a brief “lift the lip” visual inspection for early childhood caries. Cariogenic scores were shown to be positive for fourteen different children. Caries status of the incisors of eight further children could not be determined due to lack of information. Only 0.8% of infants in the United States aged 12–23 months were found to have primary anterior tooth decay after the estimates were weighted to approximate the population of infants in that age range in the United States, and 1.1% of the children could not be categorized. During conversations with one of the examiners, it became clear that there was some doubt regarding classifying particular teeth as carious. As a result, the estimated number of children that received either positive or questionable ratings on this index can be thought of as follows. The inter-examiner heterogeneity can be attributed, in large part, to a lack of proper training in recognizing carious lesions in their early stages. Additionally, It might be difficult to tell the difference between white spots caused by incipient caries and developing hypocalcifications (13).

## Conclusion

In conclusion, the white-spot lesions that are one of the earliest signs of dental caries should be taught to parents, caregivers, and doctors utilizing the lift-the-lip approach. When followed methodically, this approach not only lessens ECC but also but also reduce the parents to suffer both psychologically and financially.
